# Increased external tibial torsion is an infratuberositary deformity and is not correlated with a lateralized position of the tibial tuberosity

**DOI:** 10.1007/s00167-020-06291-z

**Published:** 2020-09-25

**Authors:** Philipp W. Winkler, Patricia M. Lutz, Marco C. Rupp, Florian B. Imhoff, Kaywan Izadpanah, Andreas B. Imhoff, Matthias J. Feucht

**Affiliations:** 1 Department for Orthopaedic Sports Medicine, Technical University Munich, Ismaninger Str. 22, 81675 Munich, Germany; 2 Department of Orthopaedics, Balgrist University Hospital, University of Zurich, Zürich, Switzerland; 3 Department of Orthopaedics and Trauma Surgery, Medical Center, Faculty of Medicine, Albert-Ludwigs-University of Freiburg, Freiburg, Germany

**Keywords:** Tibial torsion, Segmental analysis, Patellofemoral instability, Tibial tubercle, Rotational alignment, Risk factor, Torsional osteotomy

## Abstract

**Purpose:**

To perform a segmental analysis of tibial torsion in patients, with normal and increased external tibial torsion, suffering from chronic patellofemoral instability (PFI) and to investigate a possible correlation between tibial torsion and the position of the tibial tuberosity.

**Methods:**

Patients with chronic PFI who underwent torsional analysis of the lower limb using a standardized hip-knee-ankle MRI between 2016 and 2018 were included. For segmental analysis of tibial torsion, three axial levels were defined which divided the tibia into two segments: a distal, infratuberositary segment and a proximal, supratuberositary segment. Torsion was measured for the entire tibia (total tibial torsion, TTT), the proximal segment (proximal tibial torsion, PTT), and the distal segment (distal tibial torsion, DTT). Based on TTT, patients were assigned to one of two groups: Normal TTT (< 35°) or increased external TTT (> 35°). Position of the tibial tuberosity was assessed on conventional MRI scans by measuring the tibial tuberosity-trochlea groove (TT-TG) and the tibial tuberosity-posterior cruciate ligament (TT-PCL) distances.

**Results:**

Ninety-one patients (24 ± 6 years; 78% female) were included. Mean external TTT was 29.6° ± 9.1° and 24 patients (26%) had increased external TTT. Compared to patients with normal TTT, patients with increased external TTT demonstrated significantly higher values for DTT (38° ± 8° vs. 52° ± 9°; *p* < 0.001), whereas no difference was found for PTT ( – 13° ± 6° vs.  – 12° ± 6°; n.s.). Furthermore, a significant correlation was found between TTT and DTT (*p* < 0.001), whereas no correlation was found between TTT and PTT (n.s). With regard to TT-TG and TT-PCL distances, no significant differences were observed between the two groups (TT-TG: 15 ± 6 vs. 14 ± 4 mm, n.s.; TT-PCL: 22 ± 4 vs. 21 ± 5 mm, n.s.) and no correlation was found with TTT, DTT, or PTT (n.s.).

**Conclusion:**

In patients with chronic PFI, increased external TTT of greater than 35° is an infratuberositary deformity and does not correlate with a lateralized position of the tibial tuberosity.

**Level of evidence:**

Level III.

## Introduction

Alignment, tracking, and stability of the patellofemoral joint depends on complex interactions between dynamic muscle action [23], passive soft tissue restraints [32], the surface geometry of the patella and trochlea, and limb alignment [2, 12, 16, 24, 43]. Therefore, patellofemoral dysfunction, such as patellofemoral instability, is commonly seen as a multifactorial problem [24, 43].

There is growing evidence that bony geometry and limb alignment play a major role in various patellofemoral disorders. Trochlear dysplasia and a lateralized tibial tuberosity are well accepted risk factors for chronic patellofemoral instability [2, 16, 24, 29, 34, 43]. The role of valgus malalignment [8, 12, 20, 36, 48] and torsional deformities [4, 7, 11, 13, 19, 37, 42] are less understood. However, there is growing evidence that both, valgus and torsional malalignment promote patellar maltracking and instability [9, 15, 28, 30, 35]. Although associated with various patellofemoral disorders, such as anterior knee pain and patellofemoral instability [10, 11, 13, 19, 31, 37, 42], increased external tibial torsion is probably the least studied and hence least understood factor in patellofemoral dysfunction. Furthermore, little is known about the torsional geometry of the tibia [14, 18, 21, 22, 27, 38, 39, 45, 47]. From a biomechanical point of view, it is relevant whether the main torsional deformity is located proximally or distally to the tibial tuberosity. If the deformity is located proximally, increased external torsion would also result in a lateralized tuberosity and hence an increased Q angle [5]. On the other hand, a distal deformity below the tuberosity may have only little to no impact on static patellofemoral alignment.

Despite the lack of knowledge about tibial torsional geometry, osteotomies of the tibia have been described to correct external tibial torsion and good clinical results have been reported [10, 11, 13, 19, 37, 42]. Nevertheless, it remains unclear whether the osteotomy should be performed below or above the tibial tuberosity. An osteotomy performed proximal to the tibial tuberosity will also alter the position of the tuberosity. It is therefore important to precisely define the association of tibial torsion and the position of the tibial tuberosity to determine the height of torsional osteotomies.

The purpose of the present study was to conduct a segmental analysis of tibial torsion in subjects with normal and increased external tibial torsion suffering from patellofemoral instability and to investigate a possible correlation between tibial torsion and the position of the tibial tuberosity. The hypotheses were that increased external tibial torsion is an infratuberositary deformity and is not associated with a lateralized position of the tibial tuberosity.

## Material and methods

This retrospective cohort study was conducted to evaluate the torsional morphology of the tibia and its association with the position of the tibial tuberosity in patients suffering from chronic patellofemoral instability (PFI). This study was approved by the Ethics Committee of the Technical University of Munich (Nr.: 579/19 S).

Patients screened for eligibility had a history of recurrent patellar dislocations and a positive patellar apprehension test during clinical examination. Furthermore, torsional analysis of the lower limb using a standardized hip-knee-ankle MRI (1.5 T, 8-mm slice thickness) at the authors’ institution between 2016 and 2018 was required for inclusion. Torsional MRI was performed as part of a routine radiographic workup in all patients undergoing operative treatment for chronic PFI. Exclusion criteria for the present study were: skeletal immaturity, a history of lower extremity fracture or a history of surgical procedures affecting bony alignment, and presence of metal implants with artefacts on MR images or motion artifacts.

Clinical notes of all patients were reviewed to collect demographic data. All hip-knee-ankle MRI scans and conventional MRI scans were transferred from the institutions electronic picture archiving and communication system (PACS) to the OsiriX imaging software (https://www.osirix-viewer.com, Bernex, Switzerland), which was used to measure tibial torsion at different segments and lateralization of the tibial tuberosity as described in detail below. All measurements were obtained by the main observer (P.W.W.). For 20 randomly assigned patients, measurements were taken twice by the main observer (P.W.W.) at an interval of 6 weeks and once by a second observer (P.M.L.) to determine inter- and intrarater reliability.

### Segmental analysis of tibial torsion

**Fig. 1:**
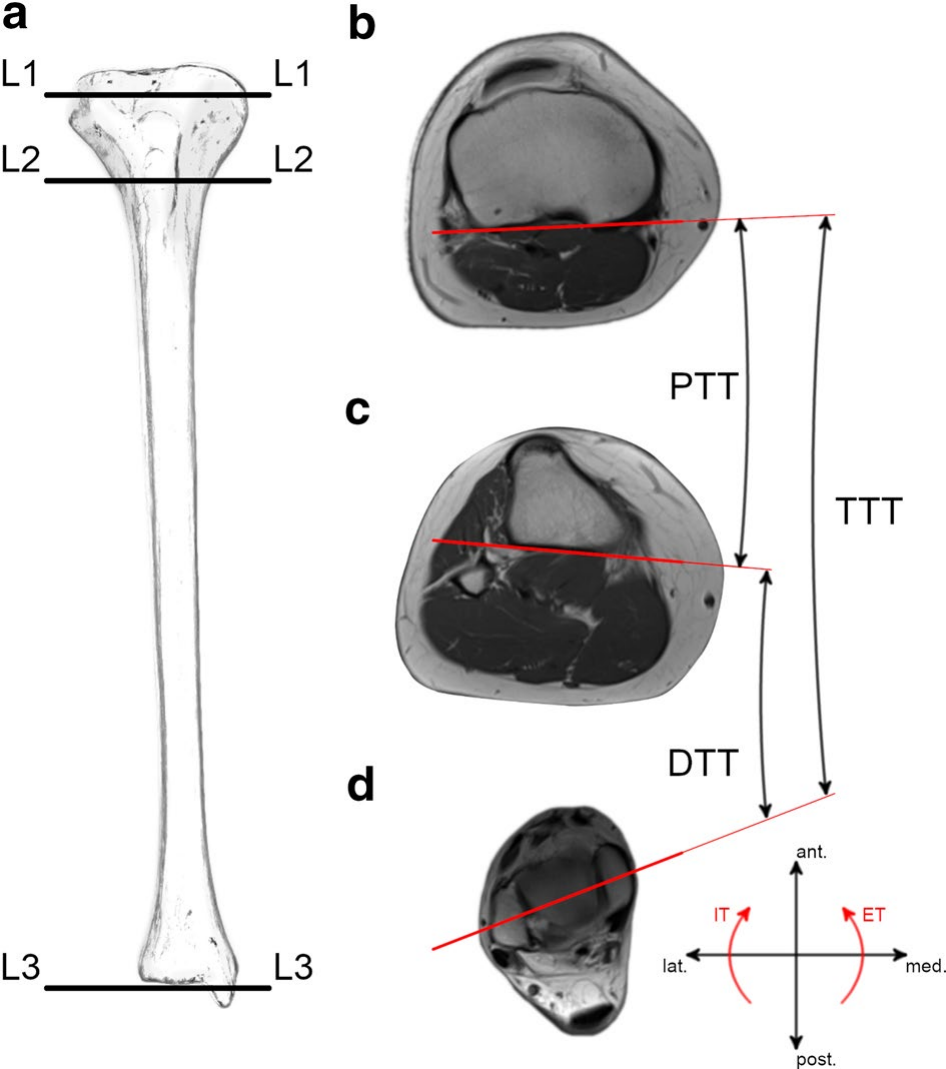
Segmental analysis of tibial torsion. **a** Schematic illustration of a right tibia showing the levels of measurement (L1, L2, L3); **b** L1: Reference line tangential to the posterior border of the tibial plateau; **c** L2: Reference line tangential to the posterior tibial cortex; **d** L3: Reference line through the centers of the medial and lateral malleolus. Total tibial torsion (TTT): angle between reference lines in L1 and L3; proximal tibial torsion (PTT): angle between reference lines in L1 and L2; distal tibial torsion (DTT): angle between reference lines in L2 and L3; ant., anterior; ET, external torsion; IT, internal torsion; lat., lateral; med., medial; post., posterior

The described method is a further development of previously established and published methods to assess tibial torsion on axial MRI scans [12, 39]. For the purpose of segmental analysis, three axial levels were defined which divided the tibia into two segments: a distal, infratuberositary segment and a proximal, supratuberositary segment (Fig. 1). Level 1 (L1) was defined as the axial slice just proximal to the apex of the fibular head [12, 39]. Level 2 (L2) was defined as the axial slice where the insertion of the patellar tendon at the tibial tuberosity could best be visualized. Level 3 (L3) was defined as the axial slice, where the talar articular surface and the medial and lateral malleolus could best be visualized [12, 39]. The proximal (supratuberositary) segment was defined as the section between L1 and L2, and the distal (infratuberositary) segment was defined as the section between L2 and L3 (Fig. 1).

Total tibial torsion (TTT) was measured between L1 and L3 as previously described [12, 39]: Reference lines were drawn in L1 tangential to the posterior border of the tibial plateau and in L3 through the centers of the medial and lateral malleolus. TTT was defined as the angle between reference lines in L1 and L3 (Fig. 1). To measure proximal (supratuberositary) and distal (infratuberositary) tibial torsion, a third reference line was drawn in L2 tangential to the posterior tibial cortex. Proximal tibial torsion (PTT) was defined as the angle between the reference lines in L1 and L2, and distal tibial torsion (DTT) was defined as the angle between the reference lines in L2 and L3. For all torsional measurements, positive values indicate external tibial torsion and negative values indicate internal tibial torsion.

Based on the amount of TTT, patients were assigned to one of two groups: Normal tibial torsion (< 35°) and increased external tibial torsion (> 35°) [45]. To date, no consensus exists for normal and pathological tibial torsion, respectively. Based on a previously published study investigating tibial torsion on 504 intact tibiae in healthy volunteers, the cut-off value from normal to increased external TTT was set at 35° [45].

### Position of the tibial tuberosity

To analyze the position of the tibial tuberosity, the tibial tuberosity-trochlea groove (TT-TG) distance, and the tibial tuberosity-posterior cruciate ligament (TT-PCL) distance were measured on conventional MRI scans, as described by Schoettle et al. [40] and Seitlinger et al. [41], respectively. The TT-TG distance was measured on axial MRI images as the mediolateral distance between the midpoint of the insertion of the patellar tendon and the trochlear groove. The TT-PCL distance was measured as the mediolateral distance between the midpoint of the insertion of the patellar tendon and the medial border of the tibial PCL attachment.

### Statistical analysis

An a priori power analysis was conducted using the free available software G*Power (Erdfelder, Faul, Buchner, Lang, HHU Düsseldorf, Düsseldorf, Germany) [17]. According to previous studies, a mean external tibial torsion of 32° and 40° can be assumed for patients suffering from patellofemoral disorders [26] and torsional malalignment syndrome [3], respectively. An effect size of 0.8 was calculated based on a standard deviation of 10° [3, 26]. An α of 0.05 and a ratio of 1:3 (increased TTT: normal TTT) resulted in a minimum total sample size of 54 subjects (13 with increased TTT, 41 with normal TTT) to achieve a statistical power of 0.8.

Statistical analysis was performed using SPSS software version 25.0 (IBM-SPSS, New York, USA). Continuous variables were calculated as mean ± standard deviation. Categorical variables were reported as count and percentages. Normal distribution of all data was evaluated with the Kolmogorov–Smirnov test. Group comparison (normal TTT vs. increased TTT) was performed with Chi-square test, Mann–Whitney *U* test, and unpaired *t* test, as appropriate. Correlation of continuous variables (TTT, PTT, DTT, TT-TG distance, and TT-PCL distance) was assessed with the Pearson correlation coefficient. The level of significance was set at *p* < 0.05.

Intraclass correlation coefficients (ICCs) were calculated to determine the intra- and interobserver reproducibility. ICC values > 0.9 were considered excellent, values between 0.8 and 0.9 were considered good and values < 0.8 were considered poor.

## Results

A total of 91 patients were included. Hip-Knee-Ankle MRI scans for segmental tibial torsion analysis were available for all patients and conventional MRI scans for TT-TG and TT-PCL distance measurements were available for 62 patients (68%).

Excellent intrarater reliability was observed for all measurements. The ICC values were 0.990, 0.981, 0.991, 0.998, and 0.998 for TT-TG, TT-PCL, TTT, DTT, and PTT, respectively. Interrater reliability was good to excellent with ICC values of 0.957, 0.833, 0.952, 0.990, and 0.983 for TT-TG, TT-PCL, TTT, DTT, and PTT, respectively.

Patient demographics and measurements of the total study group are shown in Table 1. Values for TTT were positive in all patients, indicating tibial external torsion in all patients. With regard to the different tibial segments, the values for PTT were negative in all patients (internal torsion) and the values for DTT were positive in all patients (external torsion).

**Table 1:** Patient demographics and measurements of the total study group

Age (years)	24.3 ± 6.2 (16 – 39)
*Sex*	
Male	20 (22%)
Female	71 (78%)
*Laterality*	
Right	41 (45%)
Left	50 (55%)
*Tibial torsion*	
Normal (< 35°)	67 (74%)
Increased (> 35°)	24 (26%)
Total tibial torsion (°)^a^	29.6 ± 9.1 (6.0–63.2)
Proximal tibial torsion (°)^a^	- 12.4 ± 5.6 ( – 24.9–( – 1.7))
Distal tibial torsion (°)^a^	41.7 ± 10.8 (10.1–84.4)
TT-TG distance (mm)^b^	14.4 ± 5.4 (1.9–35.6)
TT-PCL distance (mm)^b^	21.3 ± 4.5 (7.9–29.5)

Continuous variables are shown as mean ± standard deviation (range), categorical variables are shown as count and percentages

^a^Measurements were obtained in 91 patients (100%); positive values indicate external torsion; negative values indicate internal torsion

^b^Measurements were obtained in 62 patients (68%). TT-TG, tibial tuberosity-trochlea groove; TT-PCL, tibial tuberosity-posterior cruciate ligament

**Fig. 2:**
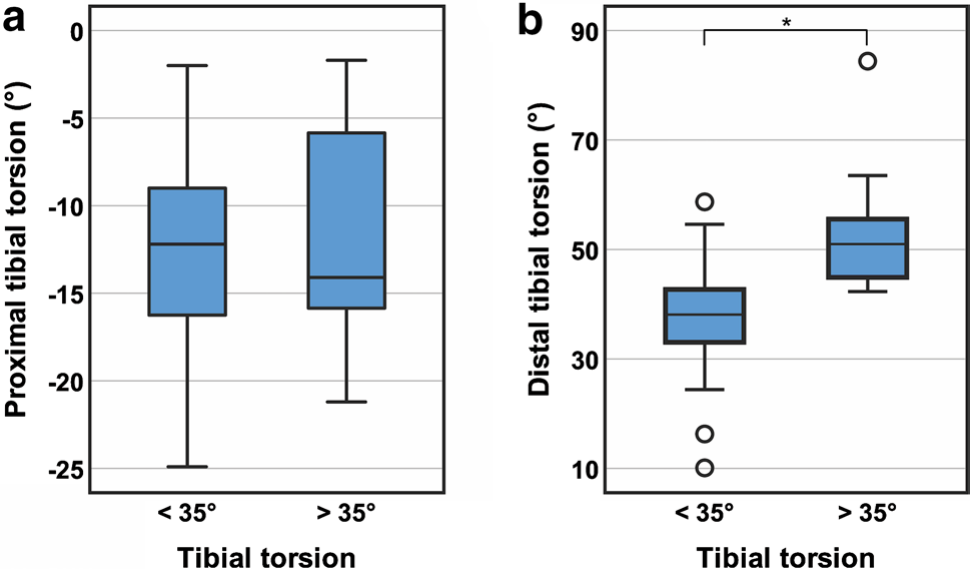
Proximal and distal tibial torsion between patients with normal (< 35°) and increased (> 35°) total tibial torsion. **a** Group comparison of proximal tibial torsion: no significant difference, n.s.; **b** group comparison of distal tibial torsion: significant difference, *p < 0.001

Increased external TTT (> 35°) was observed in 24 patients, representing 26% of the total study group. No significant differences between patients with normal and increased TTT were found for sex (n.s.), laterality (n.s.), and age (n.s.). Group comparisons of the obtained measurements are shown in Table 2. With regard to tibial torsion, patients with an increased external TTT showed a significantly higher DTT, whereas no significant difference was observed for PTT (Fig. 2). No significant difference was observed for TT-TG and TT-PCL distances (Table 2) between the groups with normal and increased TTT.

**Table 2:** Group comparison between patients with normal and increased total tibial torsion

	Tibial torsion		*p* value
	Normal (< 35°)	Increased (> 35°)	
Total tibial torsion (°)^a^	25.6 ± 6.4 (6.0–35.0)	40.8 ± 5.7 (35.8–63.2)	< 0.001 ^c^
Proximal tibial torsion (°)^a^	– 12.7 ± 5.5 ( – 24.9–(-2.0))	– 11.6 ± 5.8 ( – 21.2–( – 1.7))	n.s
Distal tibial torsion (°)^a^	37.9 ± 8.4 (10.1–58.7)	52.4 ± 9.4 (42.3–84.4)	< 0.001 ^c^
TT-TG distance (mm)^b^	14.5 ± 5.7 (1.9–35.6)	14.1 ± 4.4 (7.9–20.5)	n.s
TT-PCL distance (mm)^b^	21.5 ± 4.4 (7.9–29.5)	20.6 ± 4.8 (11.8–26.0)	n.s

Continuous variables are shown as mean ± standard deviation (range)

^a^Measurements were obtained in 91 patients (100%); positive values indicate external torsion; negative values indicate internal torsion

^b^Measurements were obtained in 62 patients (68%)

^c^Statistically significant difference between both groups (*p* < 0.05)

*TT-TG* tibial tuberosity-trochlea groove, *TT-PCL* tibial tuberosity-posterior cruciate ligament

**Fig. 3:**
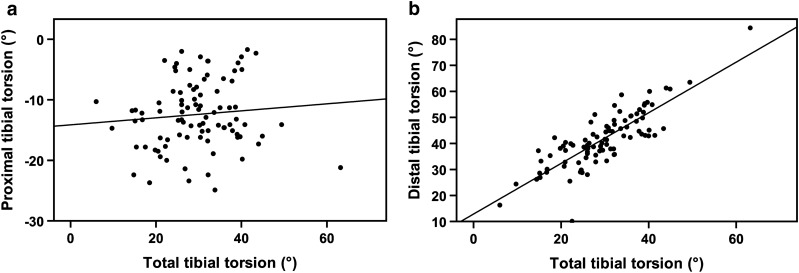
Correlation between segmental and total tibial torsion. **a** No significant correlation between total tibial torsion and proximal tibial torsion (*r* = 0.095, n.s.); **b** significant correlation between total tibial torsion and distal tibial torsion (*r *= 0.824, *p* < 0.001)

The results of the correlation analysis are shown in Table 3. A statistically significant correlation was found between TTT and DTT, whereas no significant correlation was found between TTT and PTT (Fig. 3). TTT, PTT, and DTT did not correlate with TT-TG or TT-PCL distances (Table 3).

**Table 3:** Correlation analysis (Pearson)

	Total tibial torsion	Proximal tibial torsion	Distal tibial torsion	TT-TG distance	TT-PCL distance
Total tibial torsion	–	*r* = 0.095 n.s	*r* = 0.824 *p* < 0.001^a^	*r* = – 0.119 n.s	*r* = – 0.063 n.s
Proximal tibial torsion	*r* = 0.095 n.s	–	*r* = – 0.393 *p* < 0.001^a^	*r* = 0.010 n.s	*r* = – 0.060 n.s
Distal tibial torsion	*r* = 0.824 *p* < 0.001^a^	*r* = – 0.393 *p* < 0.001^a^	–	*r* = – 0.142 n.s	*r* = – 0.027 n.s
TT-TG distance	*r* = – 0.119 n.s	*r* = 0.010 n.s	*r* = – 0.142 n.s	–	*r* = 0.511 *p* < 0.001^a^
TT-PCL distance	*r* = – 0.063 n.s	*r* = – 0.060 n.s	*r* = – 0.027 n.s	*r* = 0.511 *p* < 0.001^a^	–

*r* correlation coefficient, *p* value, *TT-TG* tibial tuberosity-trochlea groove, *TT-PCL* tibial tuberosity-posterior cruciate ligament

^a^Statistically significant correlation (*p* < 0.05)

## Discussion

The most important finding of the present study was that increased external TTT is an infratuberositary deformity. This finding was verified by two different statistical approaches: First, no significant correlation was observed between TTT and PTT, whereas a significant correlation was observed between TTT and DTT. Second, compared to patients with normal TTT (< 35°), patients with increased external TTT (> 35°) showed a significantly higher DTT, whereas no significant difference was observed for PTT. These findings indicate that increased external TTT is primarily based on torsion of the distal (infratuberositary) segment. Another important finding was that tibial torsion is not correlated with the position of the tibial tuberosity. This finding was also verified by two different statistical approaches: First, no significant correlation was observed between TTT, PTT, or DTT and TT-TG or TT-PCL distances. Second, no significant differences were found for TT-TG and TT-PCL distances between patients with normal or increased external TTT.

Back in 1909, Le Damany [33] already investigated the torsional morphology of the tibia and described a mean external tibial torsion of 23.7° based on anthropometric specimen examinations. Since then, several studies dealing with tibial torsion have been published [18, 22, 27, 38, 45, 47]. Considering the literature, a wide range of values for mean TTT becomes apparent, ranging from 20° to 41.7° [47]. A consensus exists regarding external direction of TTT [45, 47], as confirmed by the present study. The most extensive investigations for TTT analysis were performed by Strecker et al. [45] (*n* = 504) and Vanhove et al. [47] (*n* = 98), who observed a mean TTT of 34.9° ± 15.9° and 25.5° (left) / 27.7° (right), respectively. This is consistent with the findings of the present study, in which tibial torsion analysis was performed on 91 patients and revealed a mean TTT of 29.6° ± 9.1°. However, detailed information on segmental tibial torsion and its impact on distal patellofemoral alignment is currently missing. Jakob et al. [27] were the only authors so far to investigate tibial torsion in different segments. Based on six cadaveric specimens, it has been shown that the greatest increase in tibial torsion is located in the proximal quarter of the tibial bone [27], which contradicts with the findings of the present study, in which the greatest increase in tibial torsion was found distal to the tibial tuberosity. In the present study, segmental tibial torsion was assessed in 91 patients with a highly reliable measurement technique based on torsional MRI. The applied measurement technique was a further development of previously established and published methods to assess TTT on axial MRI scans [12, 39]. In contrast, the methodology of the study published by Jakob et al. exhibits some weaknesses owing to the small sample size and the lack of a precise definition of the applied levels of measurement [27].

Increased external tibial torsion, either in isolation or combined with increased femoral antetorsion, has been identified as one reason for various forms of patellofemoral dysfunction, including anterior knee pain and patellofemoral instability [5–7, 10, 11, 13, 15, 46]. Generally, tibial torsion is defined as the angulation between the most proximal and most distal part of the tibia around the longitudinal axis. Regarding the distal patellofemoral alignment, however, it is relevant whether the main torsional deformity is located proximally or distally to the tibial tuberosity. Based on the results of the present study, increased external tibial torsion can be regarded as an infratuberositary deformity. We therefore postulate that increased external tibial torsion has no relevant impact on the static distal patellofemoral alignment. This postulation is supported by a clinical study investigating the association between lower limb torsion and static patellar tracking in 59 patients with chronic PFI using torsional MR images. Although a statistically significant association between knee rotation and static patellar tracking has been demonstrated, no correlation between tibial torsion and static patellar tracking was observed [29]. Additionally, another clinical study showed no difference in tibial torsion between 30 patients with a history of patellar dislocation and an age- and sex-matched control group [12]. The distal patellofemoral alignment is influenced by the localization of the tibial tuberosity. The TT-TG and TT-PCL distances have become the most frequently used parameters to assess the position of the tibial tuberosity as indicators for distal patellofemoral realignment procedures (i.e., tibial tuberosity osteotomy) [25, 41]. It has been hypothesized that a lateralized tibial tuberosity may be caused by excessive external tibial torsion [13]. The present study, however, could not find a correlation between total or segmental tibial torsion and the TT-TG or TT-PCL distance. Furthermore, no differences in TT-TG and TT-PCL distances were observed between patients with normal or increased external tibial torsion. In accordance with other clinical studies [3, 12, 29], the present findings provide further evidence that increased external tibial torsion is not associated with a lateralized position of the tibial tuberosity.

Although increased external tibial torsion has no relevant impact on static patellofemoral alignment, it may negatively affect the gait pattern. Increased external tibial torsion has been shown to result in compensatory gait deviations with increased hip internal rotation [1]. This adaption causes the knee to rotate internally leading to a lateral directed shear force on the patella. This dynamic impact of external tibial torsion may be the explanation for the good clinical results of torsional tibial osteotomies in patients with patellofemoral dysfunction [4, 5, 11, 13, 19, 37, 42, 44]. Despite a growing number of clinical studies reporting outcomes after torsional tibial osteotomies, the level of the osteotomy (supratuberositary vs. infratuberositary) remains controversial. In the current literature, however, an osteotomy proximal to the tibial tuberosity seems to be preferred in patients with patellofemoral disorders [11, 37, 42]. It must be noted, however, that a supratuberositary torsional osteotomy will also result in medialization of the tibial tuberosity. Based on the results of the present study, which indicate that increased external tibial torsion is not associated with a lateralized tibial tuberosity, we suggest that the osteotomy height should be adjusted to the position of the tibial tuberosity: a supratuberositary osteotomy should be performed in patients with a lateralized tuberosity to prevent excessive medialization with subsequent overload of the medial patellofemoral compartment. In patients with normal position of the tibial tuberosity, an infratuberositary osteotomy should be considered. Thus, knowledge of the localization of a torsional deformity of the tibia in patients with increased external tibial torsion and chronic PFI may facilitate treatment decision-making (i.e., torsional tibial osteotomy, medialization of the tibial tuberosity).

Several limitations of this study have to be mentioned. First, there was no control group consisting of patients without patellofemoral disorders. Hip-Knee-Ankle MRI acquisition requires special indication and therefore was not economically affordable for a healthy control group. The contralateral tibiae were not investigated, because of significant physiological side-to-side differences described in the literature [38, 45, 47], which could have led to selection bias. Second, this study does not consider the dynamic behavior occurring in patients with torsional deformities. Only aspects concerning the statics of the patellofemoral joint were investigated. Third, conventional MRI was only available in 62 of the 91 patients in which the TT-TG and TT-PCL distances were measured.

## Conclusion

In patients with chronic PFI, increased external TTT of greater than 35° is an infratuberositary deformity and does not correlate with a lateralized position of the tibial tuberosity.
